# The Role of Stereotactic Frame-Based Biopsy for Brainstem Tumors in the Era of Molecular-Based Diagnosis and Treatment Decisions

**DOI:** 10.3390/curroncol29070360

**Published:** 2022-06-28

**Authors:** Yudai Hirano, Yuki Shinya, Toshiya Aono, Hirotaka Hasegawa, Mariko Kawashima, Masahiro Shin, Hirokazu Takami, Shunsaku Takayanagi, Motoyuki Umekawa, Masako Ikemura, Tetsuo Ushiku, Kazuki Taoka, Shota Tanaka, Nobuhito Saito

**Affiliations:** 1 Department of Neurosurgery, The University of Tokyo Hospital, 7-3-1 Hongo, Bunkyo-ku, Tokyo 113-8655, Japan; yuhirano-tky@umin.ac.jp (Y.H.); aono-hma@umin.ac.jp (T.A.); hirohasegawa-tky@umin.ac.jp (H.H.); mrkawashima-tky@umin.ac.jp (M.K.); takami-tky@umin.ac.jp (H.T.); takayanagi-nsu@umin.ac.jp (S.T.); umekawam-nsu@h.u-tokyo.ac.jp (M.U.); nsaito-tky@umin.net (N.S.); 2 Department of Neurosurgery, Teikyo University School of Medicine, 2-11-1 Kaga, Itabashi-ku, Tokyo 173-0003, Japan; shin.masahiro.rk@teikyo-u.ac.jp; 3 Department of Pathology, The University of Tokyo Hospital, 7-3-1 Hongo, Bunkyo-ku, Tokyo 113-8655, Japan; ikemuram-pat@h.u-tokyo.ac.jp (M.I.); usikut-tky@umin.ac.jp (T.U.); 4 Department of Hematology and Oncology, The University of Tokyo Hospital, 7-3-1 Hongo, Bunkyo-ku, Tokyo 113-8655, Japan; taokak-int@h.u-tokyo.ac.jp

**Keywords:** stereotactic biopsy, brainstem tumor, frame-based

## Abstract

Stereotactic frame-based brain tumor biopsy (SFB) is a potent diagnostic tool considering its minimal invasiveness, though its diagnostic power and safety for brainstem lesions remain to be discussed. Here, we aimed to examine the usefulness of SFB for brainstem tumors. Twenty-two patients with brainstem tumors underwent 23 SFBs at our institution during 2002–2021. We retrospectively analyzed patient characteristics, tumor pathology, surgical procedures, and outcomes, including surgery-related complications and the diagnostic value. Seven (32%) tumors were located from the midbrain to the pons, eleven (50%) in the pons only, and four (18%) from the pons to the medulla oblongata. The target lesions were in the middle cerebellar peduncles in sixteen procedures (70%), the cerebellum in four (17%), the inferior cerebellar peduncles in two (9%), and the superior cerebellar peduncles in one (4%). A definitive diagnosis was made in 21 patients (95%) at the first SFB. The diagnoses were glioma in seventeen (77%) cases, primary central nervous system lymphoma in four (18%), and a metastatic brain tumor in one (5%). The postoperative complications (cranial nerve palsy in three [13%] cases, ataxia in one [4%]) were all transient. SFB for brainstem tumors yields a high diagnostic rate with a low risk of morbidity.

## 1. Introduction

Brainstem tumors account for approximately 10% of all primary central nervous system tumors, with the most common diagnoses being gliomas and primary central nervous system lymphomas (PCNSLs) [1,2,3,4]. Gliomas and PCNSLs arising in the brainstem tend to be highly malignant and have a poor prognosis. Resection of these tumors is not feasible because of the surrounding important anatomical structures and the infiltrative nature of the tumor. Therefore, drug therapy with or without radiation is the choice of treatment, which undoubtedly requires accurate diagnosis. One of the biggest issues is that their radiological diagnosis may not always be reliable even with modern imaging modalities; thus, pathological diagnosis based on tumor specimens is desirable to determine the appropriate treatment strategies [5]. In addition, molecular diagnosis with technologies such as next-generation sequencing are also crucial for World Health Organization (WHO) classification and prognostication of brainstem gliomas [6]. It is possible to make a definitive diagnosis and predict the prognosis based on the identification of gene mutations in tumors by obtaining specimens. Moreover, precision medicine, which targets the mutations based on cancer multi-gene panel testing, can be performed, and epigenetic analysis and transcriptome analysis possibly may lead to the development of new treatment methods in the future. Therefore, safe and reliable surgical techniques for the location of difficult-to-access lesions, such as those in the brainstem region, are becoming increasingly important. However, conventional open biopsies of brainstem tumors require invasive skull-base approaches which may result in postoperative neurological decline and other non-neurological complications, potentially hindering the introduction of subsequent essential treatment.

Stereotactic frame-based brain tumor biopsy (SFB) is a modern surgical technique that enables highly accurate targeting through a burr hole guided by high resolution magnetic resonance imaging (MRI) taken under rigid head fixation. To date, numerous studies, including meta-analyses, have revealed the efficacy and safety of SFB [7,8,9]; however, there are only a few studies on brainstem tumors, and the safety and efficacy of SFB for such tumors remain unclear. In 2002, our institution initiated a multidisciplinary approach including SFB-based collection of tumor specimens for brainstem lesions. In this study, we aimed to verify the diagnostic validity and safety of SFB. In our principal conclusion, SFB for brainstem tumors yields a high diagnostic rate (95%) and low morbidity (17%; all were transient symptoms) in our study.

## 2. Materials and Methods

### 2.1. Study Population and Data Collection

This retrospective observational study included the patients with brainstem lesions who underwent SFB at our hospital from 2002 to 2021. SFB was indicated for the patients with brainstem tumors suspected to be malignant based on their clinical course and imaging findings. The patients who could not tolerate general anesthesia due to their exceedingly poor general condition were not eligible for the surgery. Biopsies from small, confined lesions inside the brainstem were avoided, and lesions extending to the cerebellar peduncle were the primary targets. The data were collected from an institutional database. Twenty-three SFBs were performed for the 22 patients. All patients provided written informed consent for participation, and the study was approved by our institutional review board (Number 2231 and G10028).

### 2.2. Surgical Setting and Procedures

**Figure 1:**
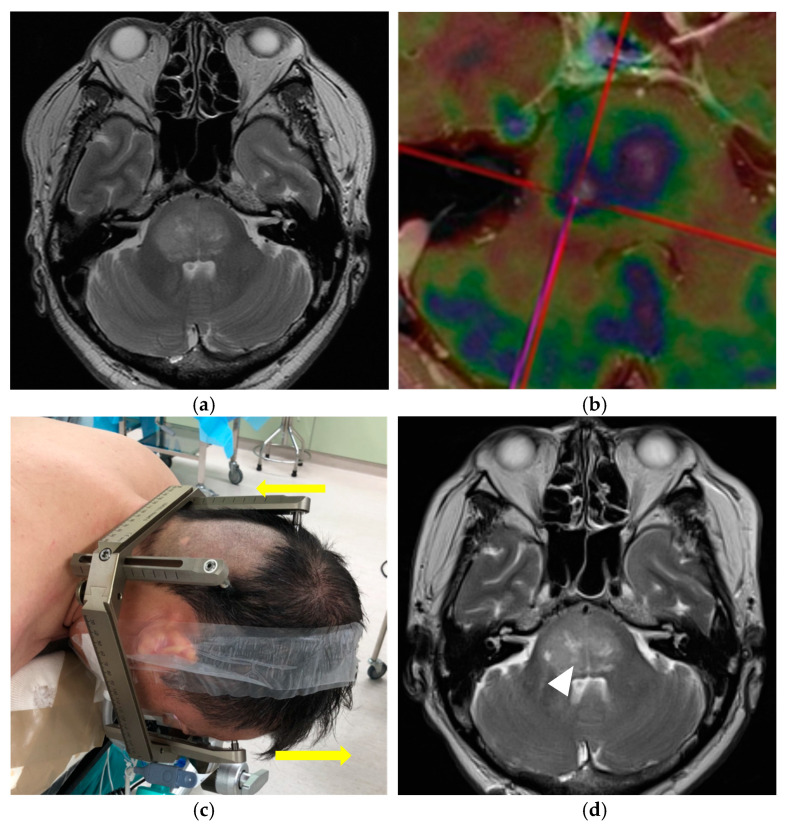

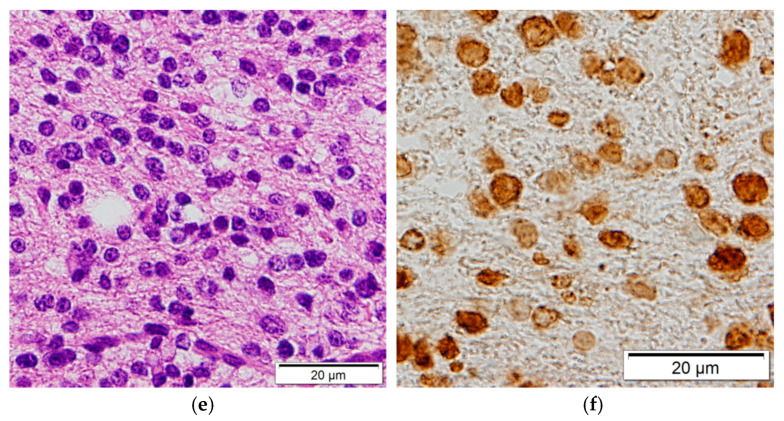
A 60-year-old man presented with headache, diplopia, and ataxia (Case 18). (**a**,**b**) Magnetic resonance imaging (MRI) and positron emission tomography (PET) revealed a pontine tumor extending also to the right thalamus, bilateral medial temporal lobe, right mesencephalic tegmentum, medulla oblongata, and bilateral cerebellum (maximum diameter: 37 mm, tumor volume: 17.8 mL); the surgical trajectories are set through the right middle cerebellar peduncle to the brainstem tumor, aiming for postoperative neurological preservation by using the FrameLink software installed in StealthStation S7 surgical navigation system (Medtronic plc); (**c**) Surgical setup for stereotactic frame-based biopsy (SFB) of a brainstem tumor. The physical characteristics of this patient are a large head size, short neck, and well-developed occipital muscles. The frame was fixed at four points on the forehead and back of the head by shifting the frontal pins slightly upward from the orbitomeatal baseline (arrow), to reduce the possibility of interference with the surgical field, which comprised a skin incision and burr hole; (**d**) No hemorrhage was observed on the MRI performed within 24 h after surgery. The tumor specimen was obtained from the targeted location (right middle cerebellar peduncle; arrowhead); (**e**) Histologic features of a diffuse midline glioma. Hematoxylin and eosin staining showing the diffuse growth of atypical glia cells with round nuclei; (**f**) Immunohistologic staining showing that the atypical cells are positive for H3K27M.

Before surgery, contrast-enhanced computed tomography (CT) and MRI were routinely performed, and nuclear medical examinations were performed via ^18^F-fluorodeoxyglucose positron emission tomography (^18^F-FDG PET) and single-photon emission computed tomography (SPECT), as needed to improve the accuracy of diagnosis (Figure 1a,b). Patients were fixed with a Leksell Coordinate Frame (Elekta AB, Stockholm, Sweden) under local anesthesia. The frame was fixed at four points on the forehead and the occiput. There were two important contrivances in the head fixation to facilitate the procedures. First, to fully expose the suboccipital area in which the burr hole would be placed, the posterior bar needed to be placed as low as possible while the occipital pins were placed above the superior nuchal line. Second, to leave the airway open for the anesthesia management, the frontal bar was placed slightly above the tip of the nose (Figure 1c). The head fixation was performed in the same fashion both for the adult and pediatric patients, but with caution, especially in the pediatric patient, due to the variability in the thickness of the developing skull. Subsequently, stereotactic, contrast-enhanced, thin-slice CT or contrasted time-of-flight MRI with a slice thickness of 1 mm was performed to safely and accurately define the surgical trajectory for collection of the tumor specimen. For patients with less-contrast-enhanced tumors, the tumor target was defined with a combination of thin-slice fluid-attenuated inversion recovery (FLAIR), T2-weighted MRI, PET, and/or SPECT images. As per our policy, the stereotactic CT is performed for pediatric cases under 12 years old instead of MRI to shorten the examination time with the fixation, considering the risk of body movement during the imaging examination. In principle, the target is set at the site of a well-enhanced lesion that extends to the cerebellar peduncle. When the tumor has spread extensively, the highly active component can be determined by PET and/or SPECT. During preoperative planning, we used the infratentorial transcerebellar approach with the patient in the prone position for all cases. The surgical trajectories were set through the cerebellar peduncle to the brainstem tumor, aiming for postoperative neurological preservation by using the FrameLink software (Elekta AB) (Figure 1d). Specifically, we avoided the nerve nuclei and tracts and confirmed that there were no arteries, veins, or cerebellar sulci along the trajectory.

Patients were placed in the prone position under general anesthesia. After draping, a bilateral arc support with a slide was fixed to the frame, and the Leksell Multipurpose Stereotactic Arc was attached to the arc support and adjusted with the arc angle. The operator routinely verified the coordinates at each step. A 4 cm-long linear skin incision was made just above the puncture point, and a burr hole was made with a high-speed drill. After a dural incision was made, we confirmed that there were no major vessels on the cerebellar surface. After aligning the arc, the needle (typically a 2.1 mm-wide puncture needle) was slowly inserted to the target. Then, the side wall of the needle was opened, and a 2.5 mL syringe attached onto the needle was gently pulled. While maintaining appropriate suction force, the inner needle was rotated so the tumor specimens was bitten off. The inner needle was then removed, and the lumen was flushed to collect the tumor specimens. This procedure was repeated four times while rotating the biopsy needle by 90°. During surgery, an intraoperative pathology examination was routinely performed to see if the specimens were suitable for detailed pathological evaluation. After hemostasis was confirmed, the wound was closed.

A CT was performed immediately after surgery to assess hemorrhagic complications, and an MRI was performed on postoperative day 1 to confirm the actual target. According to the final histological diagnosis, appropriate treatment was initiated.

## 3. Results

### 3.1. Patient Characteristics

The baseline characteristics of the entire cohort are summarized in Table 1. The median age at the time of surgery was 38 years (range 3–70 years), with a median postoperative follow-up period of 18 months (range 1–88 months). A total of seven (32%) tumors were from the midbrain to the pons, eleven (50%) in the pons only, and four (18%) from the pons to the medulla oblongata. For stereotactic imaging, a thin-slice MRI was performed in all but two pediatric patients in whom CT was alternatively used. The actual targets were set in the middle cerebellar peduncles in sixteen procedures (70%), the cerebellum in four (17%), the inferior cerebellar peduncles in two (9%), and the superior cerebellar peduncles in one (4%). A definitive diagnosis was made for 21 patients (95%) at the first SFB. The remaining one patient (5%) required a second SFB because severe necrotic changes in the specimens obtained at the first SFB made a definitive diagnosis difficult.

### 3.2. Histopathological Diagnosis and Postoperative Course

The histological diagnoses included glioma in seventeen (77%) cases, diffuse large B-cell lymphoma in four (18%), and metastatic lung carcinoma in one (5%). The postoperative complications included transient cranial nerve palsy in three (13%) cases (two cases of abducens nerve palsy and one of facial nerve palsy) and transient ataxia in one (4%) case, and no permanent complications were observed. All patients subsequently received radiation and/or chemotherapy. Radiotherapy was performed in all cases, and chemotherapy was performed in 20 (91%) cases; the remaining two patients had a poor general condition. An illustrative case is provided in Figure 1.

## 4. Discussion

This study demonstrated that SFB can be a safe method for performing biopsies of brainstem tumors, even though the targets and trajectories are limited. Our results were satisfactory, with a diagnostic rate of 95% at the first attempt and 100% after two attempts. The reason why the diagnostic accuracy was high in this cohort is that the sensitivity was increased by integrating the nuclear medicine examination in almost all cases. In addition, surgical complication rates, such as permanent neurological deficits and postoperative hemorrhage, were remarkably low.

In previous reports, the diagnostic rate of brainstem tumor biopsy varied from 84% to 97% [7,8,9,10,11,12,13,14,15], which are comparable to our results. Regarding the approach, the transfrontal and transcerebellar routes are the two major surgical routes [11,15,16,17]; however, there is a paucity of evidence as to which approach is more suitable [13,14,18]. Dellaretti et al. [12] reported that the transfrontal approach achieved a higher diagnostic rate (95%) than the transcerebellar approach (84%), but the difference was not statistically significant, similar to the observation in other studies [19,20]. For pediatric brainstem tumors, the diagnostic rate via the transcerebellar approach was 96% [13]. In our cohort, a high diagnostic rate and a low complication rate were both achieved, indicating that the transcerebellar approach is safe, though it requires ingenuity to target the accessible lesions such as those in the cerebellar peduncle. Furthermore, since the trajectory is shorter than the transfrontal approach, the transcerebellar peduncle route is likely to be more beneficial. It would be desirable to select the optimal approach based on the maturity of the surgical procedure at each facility and the lesion location. Furthermore, the routine integration of nuclear medicine examinations such as PET and SPECT to set the target site may have optimized and improved the diagnostic rate. Although some cases may be diagnosed as non-neoplastic lesions as a result of SFB, in this series of SFB for brainstem lesions, all specimens were neoplastic lesions, leading to appropriate radiation and/or chemotherapy.

In previous reports, the rate of major complications or permanent disability owing to SFB was approximately 0.5–3% [7,8,9,10,17,18], and there was no death, postoperative hemorrhage, or permanent morbidity in our series, indicating greater safety than has been reported in previous studies. Complications can be reduced by careful examination of preoperative images for, for instance, the presence of blood vessels in the trajectory, and to avoid eloquent areas as the targets. Furthermore, we have devised ways to reduce the amount of tumor specimens as much as possible by selecting narrow puncture needles and limiting the tumor collection. On the other hand, reducing the amount of the sample collected may reduce the diagnostic rate. Our method has balanced the diagnostic yield and the safety. Labuschagne et al. recently described their techniques of intraoperative neurostimulation aiming for the prevention of accidental biopsy of eloquent areas [21], which seems to offer an appealing means of further improving the safety of this method. In addition, stereotactic biopsy in the semi-sitting position under local anesthesia is also a candidate with further minimal invasiveness.

Other than SFB, Peciu-Florianu et al. recently introduced frameless robot-assisted stereotactic biopsy, demonstrating a diagnostic rate of 96% and a permanent complication rate of 2.9%. [10]. Carai et al. also reported their experience in robot-assisted stereotactic pontine biopsy in a series of diffuse intrinsic pontine glioma pediatric patients as a safe procedure with a high diagnostic rate [22]. This method not only appears to be as effective as SFB but is also advantageous because it is free from preoperative frame fixation which is somewhat laborious and may limit surgical entry. Alternatively, the accuracy of frameless stereotactic biopsy may be enhanced by the use of intraoperative MRI and could be applicable for challenging lesions such as brainstem tumors [23]. Accumulation of evidence is needed to further clarify the role of frameless robot-assisted stereotactic biopsy. 

The main limitations of this study were its retrospective nature and the small sample size. Further case accumulation is needed to reach more robust conclusions. By targeting the middle cerebellar peduncle and reducing the amount of sample to be collected, it was possible to ensure safety while maintaining a high diagnostic rate. On the other hand, considering the intra-tumoral heterogeneity, the target specimen does not always represent the entire tumor, which is a common problem with all needle biopsies. We strive to avoid underestimating the tumor activity by performing nuclear medicine tests. The transcerebellar SFB performed at our institution is a safe and effective method, and is one of the options of choice for undiagnosed brain stem lesions.

## 5. Conclusions

The present study demonstrated that SFB for brainstem tumors is a minimally invasive and safe surgical technique, yielding a high diagnostic rate and low morbidity.

## Figures and Tables

**Table 1:** Summary of stereotactic tumor biopsies of neoplastic lesions in the brainstem.

No.	Age, Sex	Tumor Location	Target	Diagnosis	Stealth Imaging	PET/SPECT	Follow-Up	Latest mRS	Complications	Postoperative Treatment
1	3 y, F	Pons	Middle CP	Astro, IDH-mt, Gr2	CT	Yes	33 months	5	Facial nerve palsy (transient)	Chemo, RT
2	6 y, F	Midbrain Pons Medulla oblongata	Middle CP	GBM	CT	No	1 month	6	None	RT
3	11 y, F	Midbrain Pons Cerebellum	Middle CP	Astro, IDH-mt, Gr2	CT MRI	Yes	9 months	6	None	Chemo, RT
4	13 y, M	Pons	Middle CP	Astro, IDH-mt, Gr3	MRI	Yes	12 months	6	None	Chemo, RT
5	21 y, M	Midbrain Pons Medulla oblongata Cerebellum	Middle CP	Astro, IDH-mt, Gr3	MRI	Yes	29 months	6	None	Chemo, RT
6	22 y, M	Pons Cerebellum	Middle CP	Astro, IDH-mt, Gr2	MRI	No	61 months	6	None	Chemo, RT
7	28 y, M	Pons	Inferior CP	Astro, IDH-mt, Gr3	MRI	Yes	39 months	6	None	Chemo, RT
8	34 y, M	Midbrain Pons Cerebellum	Middle CP	Astro, IDH-mt, Gr3	MRI	Yes	17 months	5	None	Chemo, RT
9	34 y, M	Pons Cerebellum	Middle CP	Metastatic carcinoma	MRI	Yes	1 month	5	Abducens nerve palsy (transient)	RT
10	34 y, M	Pons	Middle CP	DMG H3 K27-altered	MRI	Yes	39 months	1	None	Chemo, RT
11	38 y, F	Pons Cerebellum	Cerebellum	PCNSL	MRI	Yes	19 months	6	None	Chemo, RT
12	38 y, M	Pons	Inferior CP	Astro, IDH-mt, Gr2	MRI	Yes	86 months	2	Diplopia (transient)	Chemo, RT
13	39 y, F	Pons Cerebellum	Middle CP	Astro, IDH-mt, Gr3	MRI	Yes	8 months	N/A	None	Chemo, RT
14	47 y, F	Pons Cerebellum	Middle CP	DMG H3 K27-altered	MRI	Yes	7 months	6	None	Chemo, RT
15	47 y, M	Pons Cerebellum	Middle CP	Astro, IDH-mt, Gr2	MRI	Yes	28 months	2	None	Chemo, RT
16	48 y, M	Midbrain Pons Cerebellum	Middle CP	Astro, IDH-mt, Gr3	MRI	Yes	16 months	4	None	Chemo, RT
17	54 y, F	Pons	Cerebellum	PCNSL	MRI	Yes	13 months	6	None	Chemo, RT
18	60y, M	Midbrain Pons Medulla oblongata Cerebellum	Middle CP	DMG H3 K27-altered	MRI	Yes	36 months	5	None	Chemo, RT
19-1	62 y, M	Pons Cerebellum	Cerebellum	N/A	MRI	Yes	6 months	N/A	None	Chemo, RT
19-2	62 y, M	Pons Cerebellum	Superior CP	GBM	MRI	Yes	6 months	6	Ataxia (transient)	Chemo, RT
20	65 y, F	Pons Medulla oblongata Cerebellum	Cerebellum	PCNSL	MRI	Yes	88 months	1	None	Chemo, RT
21	70 y, F	Midbrain Pons Cerebellum	Middle CP	PCNSL	MRI	No	22 months	6	None	Chemo, RT
22	70 y, M	Pons Cerebellum	Middle CP	Astro, IDH-mt, Gr2	MRI	Yes	9 months	1	None	Chemo, RT

Astro: astrocytoma, CP: cerebellar peduncle, CT: computed tomography, DMG: diffuse midline glioma, F: female, GBM: glioblastoma, Gr: grade, M: male, MRI: magnetic resonance imaging, mRS: modified Rankin Scale, mt: mutant, N/A: not available, PCNSL: primary central nervous system lymphoma, PET: positron emission tomography, RT: radiation therapy, SPECT: single-photon emission computed tomography.

## Data Availability

Anonymized data in this article will be available by request from any qualified investigator, and information about the method of analysis will be available from the corresponding author upon reasonable request.
